# Molecular glue degraders versus PROTACs: a novel paradigm in targeted protein degradation

**DOI:** 10.1097/MS9.0000000000005002

**Published:** 2026-04-22

**Authors:** Aiman Tahir, Sarum Ali Khan, Eashaal Imtiaz, Muhammad Adnan Qamar

**Affiliations:** a Department of Medicine, Azad Jammu and Kashmir Medical College Muzaffarabad, Azad Jammu & Kashmir, Pakistan; b Department of Medicine, Wah Medical College, Wah Cantt, Pakistan; c Department of Medicine, Ayub Medical College Abbottabad, Abbottabad, Pakistan; d Department of Internal Medicine, Spinghar Medical University, Kabul, Afghanistan

**Keywords:** BTK, cancer therapeutics, E3 ubiquitin ligase, hook effect, IKZF1/3, molecular glue degraders, PROTACs, targeted protein degradation, ubiquitin-proteasome system, undruggable proteome

## Abstract

Many proteins that cause cancer are still out of reach for regular drugs because they do not have the right structure for small molecules to bind to. Targeted protein degradation (TPD) is a new way to do things. Instead of blocking these proteins, it takes over the cell’s own disposal system, the ubiquitin-proteasome system, to get rid of them completely. Two main strategies have emerged: molecular glue degraders, which are small compounds that help an E3 ligase find and tag a target protein, and PROteolysis TArgeting Chimeras (PROTACs), which are engineered bifunctional molecules that physically connect a target to a degradation pathway. Both have shown real promise in early clinical work. PROTACs that target Bruton’s tyrosine kinase have shown great response rates in B-cell cancers, and molecular glues that target Ikaros family proteins (IKZF1/3) are still helping people with blood cancers. But there are still problems. PROTACs have trouble dissolving and getting into the body through the mouth. The “hook effect” can make them less effective at higher doses, and both strategies rely heavily on a small number of E3 ligases. To make progress, we need to add more enzymes to our toolkit, design molecules better, and get better data from clinical trials. With cooperation from academia, industry, and regulators, TPD could significantly expand the druggable proteome and transform cancer treatment.

*To the Editor,*

Despite significant advances in precision oncology, a considerable proportion of disease-driving proteins remain therapeutically “undruggable,” continuing to limit effective cancer treatment. Many oncogenic regulators, particularly transcription factors and scaffolding proteins, lack well-defined ligand-binding pockets required for conventional small-molecule inhibition, allowing malignant signaling to persist even in the presence of targeted therapies. Targeted protein degradation (TPD) has emerged as a promising pharmacological strategy that shifts the focus from inhibition toward elimination of the protein itself. By harnessing the ubiquitin-proteasome system, this approach enables selective degradation of pathogenic proteins and offers a way to overcome some of the limitations of traditional drug design. Given that cancer accounts for nearly 10 million deaths annually, expanding the druggable proteome remains an urgent priority. In this context, two major TPD strategies – molecular glue degraders and PROteolysis TArgeting Chimeras (PROTACs) – have attracted considerable attention for their therapeutic potential^[^1,2^]^.

Molecular glue degraders are typically small, monovalent compounds that promote or stabilize interactions between an E3 ubiquitin ligase and a target protein, thereby inducing proximity-driven ubiquitination without requiring a pre-existing binding pocket. In contrast, PROTACs are heterobifunctional molecules composed of two ligands – one targeting the protein of interest and the other recruiting an E3 ligase – connected by a chemical linker, which enables formation of an a ternary complex and subsequent protein degradation. These differences in mechanism are reflected in their pharmacological behavior: molecular glues generally exhibit more favorable pharmacokinetic properties owing to their smaller size, whereas PROTACs provide greater flexibility and can, in principle, be adapted to a wider range of protein targets^[^3^]^.

Emerging evidence from translational and early clinical studies suggests that both approaches can achieve meaningful target depletion, even in challenging oncogenic settings. Several PROTACs have progressed into early-phase clinical trials, including agents targeting Bruton’s tyrosine kinase (BTK) in relapsed or refractory B-cell malignancies, where response rates approaching 80–90% have been reported in selected cohorts. PROTACs directed against transcriptional regulators such as STAT3 have also shown encouraging activity in lymphoma. Molecular glues, particularly those targeting Ikaros family proteins (IKZF1/3), continue to demonstrate clinical benefit in hematologic malignancies, with generally acceptable safety and tolerability profiles. Taken together, these findings suggest that TPD strategies may help address resistance mechanisms associated with conventional inhibitors. However, most of the available data remain derived from early-phase studies with relatively small sample sizes, and longer-term efficacy and optimal patient selection still need to be clarified^[^4^]^.

Despite these advances, a number of challenges remain. PROTACs are often limited by their physicochemical properties, including poor solubility and suboptimal oral bioavailability. A “hook effect” at higher concentrations has also been described, which may reduce degradation efficiency. In addition, both molecular glues and PROTACs depend on a relatively limited set of well-characterized E3 ligases, which restricts the range of targetable proteins and may contribute to resistance or off-target effects. The complexity and cost of design and manufacturing – particularly in the case of PROTACs – may also limit accessibility, especially in resource-constrained settings. Furthermore, the long-term biological consequences of sustained protein degradation are not yet fully understood, underscoring the need for careful clinical evaluation^[^2,5^]^.

In summary, molecular glue degraders and PROTACs represent two complementary approaches within the evolving field of TPD. Future efforts should focus on expanding the diversity of E3 ligases, refining design strategies – particularly for molecular glues – and generating more robust clinical evidence through well-designed studies. Closer collaboration between academia, industry, and regulatory bodies will be important to accelerate clinical translation and improve accessibility in the years ahead.

## Data Availability

All data used is accessible.
